# Supplementary material for: Habitat occupancy of the threatened Diademed Plover (Phegornis mitchellii) is not affected by llama grazing or peatland size, but declines with peatland humidity
Source: PLoS One. 2024 Jul 11;19(7):e0305462. doi: 10.1371/journal.pone.0305462 (PMC11239070; doi:10.1371/journal.pone.0305462)
Supplement: S1 Appendix — (DOCX) [file pone.0305462.s001.docx]

**S1 Appendix**

**
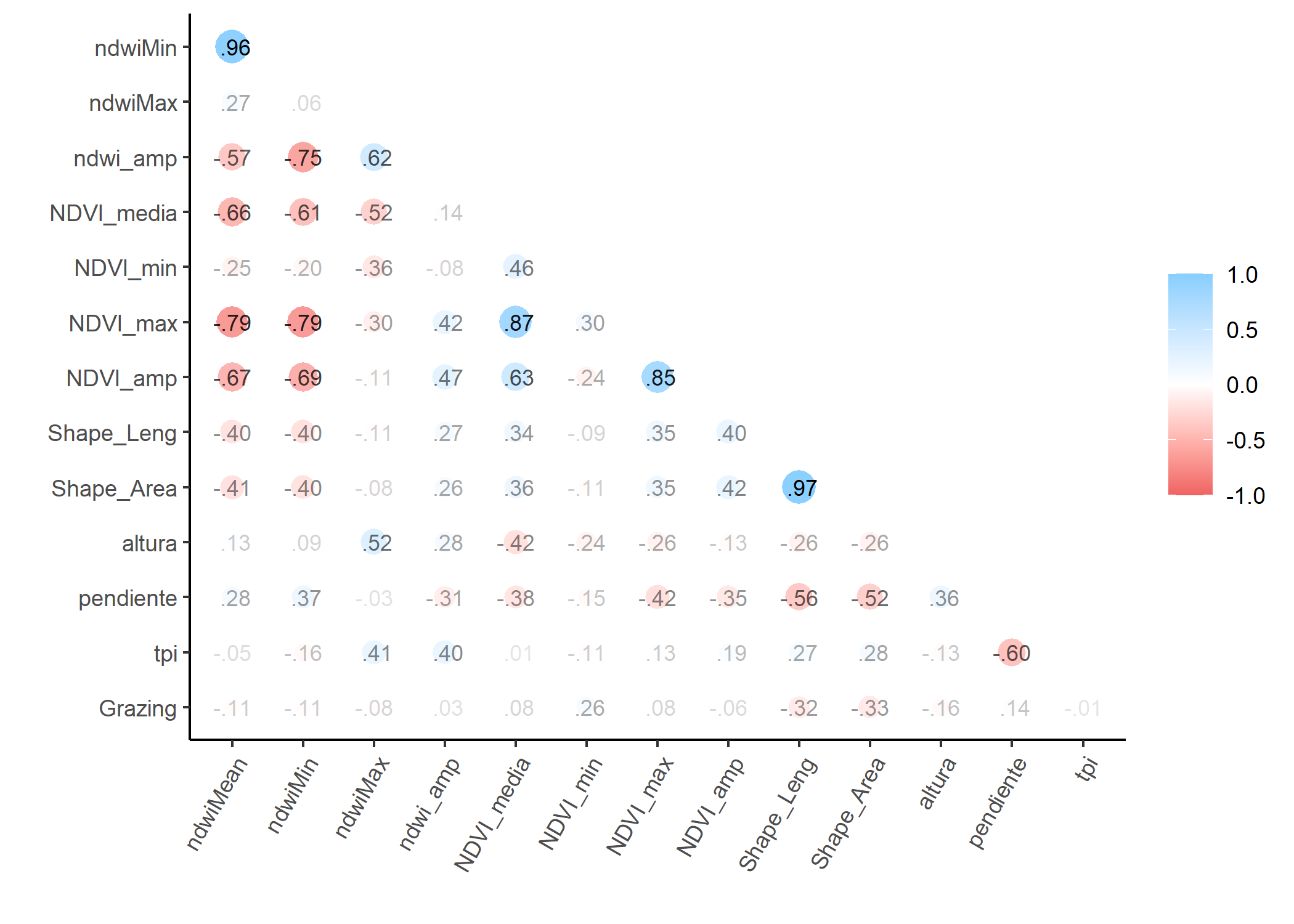
**

**Fig 1.** Correlation plot among variables. tpi: Topographic index, altura: Height, pendiente: slope, NDVI_media: mean NDVI.

| **Model** | **elPD** | **PD** | **WAIC** |
| --- | --- | --- | --- |
| Psi(NDWI, slope) p(area) | -35.5 | 4.06 | 79.35 |
| Psi(NDWI) p(.) | -36.91 | 2.99 | 79.83 |
| Psi(NDWI , slope) p(.) | -36.67 | 3.96 | 81.3 |
| Psi(NDWI) p(area) | -36.7 | 4.19 | 81.78 |
| Psi(NDWI, grazing) p(.) | -37.02 | 4.1 | 82.02 |
| Psi(area, NDWI) p(.) | -36.91 | 4.43 | 82.47 |
| Psi(area , NDWI) p(area) | -36.27 | 5.16 | 82.88 |
| Psi(NDWI , grazing) p(area) | -36.78 | 5.22 | 84.04 |
| Psi(area) p(.) | -39.89 | 2.81 | 85.41 |
| Psi(area) p(area) | -38.83 | 4.21 | 86.05 |
| Psi(grazing) p(area) | -39.95 | 3.17 | 86.44 |
| Psi(slope, grazing) p(area) | -39.55 | 3.57 | 86.16 |
| Psi(area*grazing) p(.) | -38.28 | 4.8 | 86.31 |
| Psi(area*grazing) p(area) | -37.75 | 5.54 | 86.6 |
| Psi(slope, area) p(.) | -39.96 | 3.41 | 86.75 |
| Psi(area, grazing) p(.) | -39.77 | 3.81 | 87.17 |
| Psi(.)p(area) | -40.6 | 3.21 | 87.5 |
| Psi(slope) p(area) | -40.36 | 3.67 | 87.9 |
| Psi(slope , area) p(area) | -38.84 | 5.14 | 87.96 |
| Psi(area , grazing) p(area) | -38.61 | 5.34 | 87.8 |
| Psi(.)p(.) | -42.16 | 1.95 | 88.23 |
| Psi(slope) p(.) | -42.14 | 2.94 | 90.18 |
| Psi(grazing) p(.) | -42.26 | 2.89 | 90.31 |
| Psi(slope, grazing) p(.) | -42.3 | 3.7 | 92.03 |

**Table S1**. The 24 non-spatial models ranked by WAIC. elPD is the expected log pointwise predictive density and PD is the number of effective parameters estimated for each model.
