# Supplementary figures and images for: Habitat occupancy of the threatened Diademed Plover (Phegornis mitchellii) is not affected by llama grazing or peatland size, but declines with peatland humidity
Source: PLoS One. 2024 Jul 11;19(7):e0305462. doi: 10.1371/journal.pone.0305462 (PMC11239070; doi:10.1371/journal.pone.0305462)

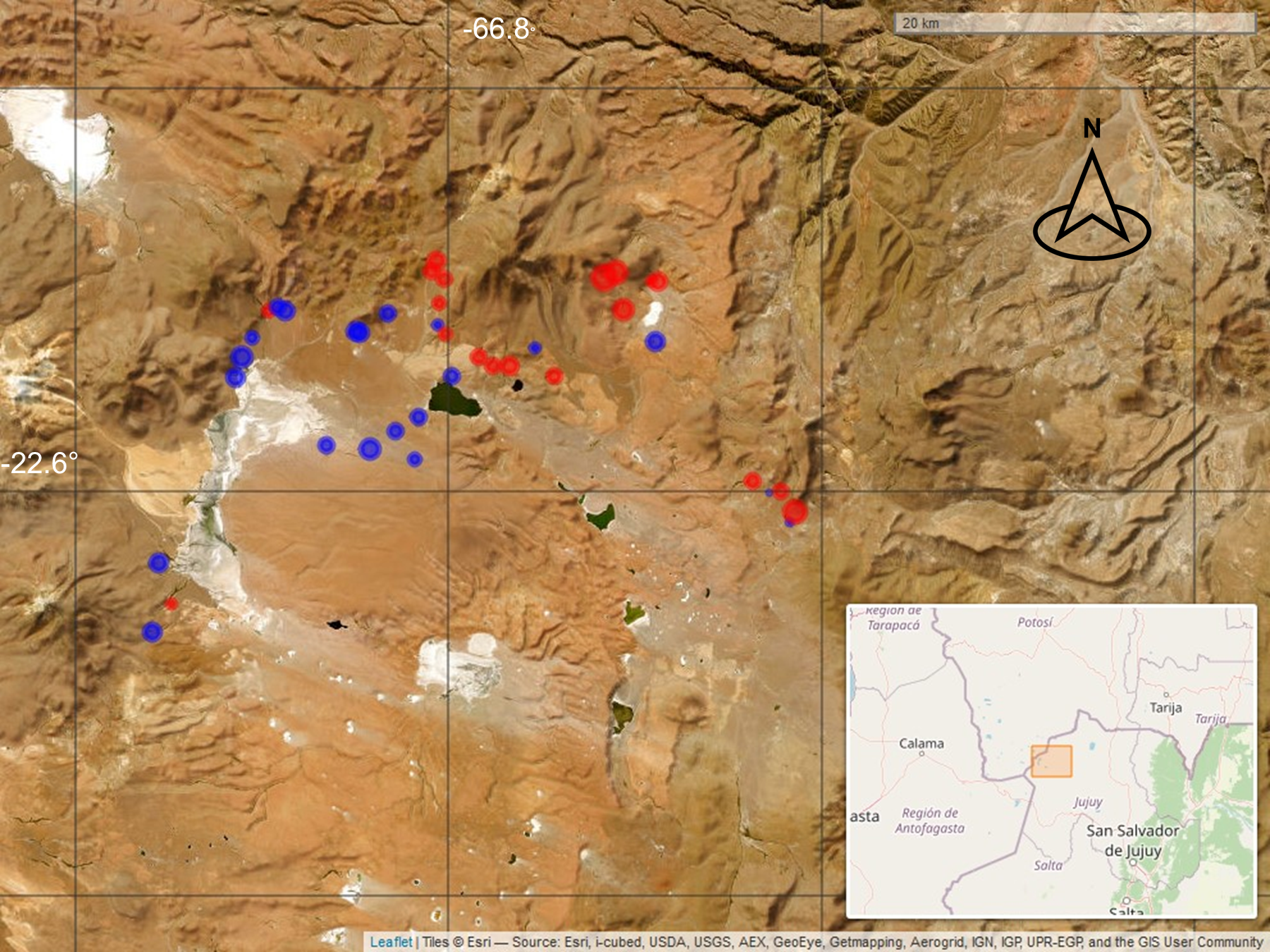

Supplement: S1 Fig — Each dot represents a surveyed peatland. Red dots indicate positive spatial random errors, blue dots negative. The size of the dot is proportional to the magnitude of the spatial error. (TIF) [file pone.0305462.s002.tif]
