# Supplementary material for: Habitat occupancy of the threatened Diademed Plover (Phegornis mitchellii) is not affected by llama grazing or peatland size, but declines with peatland humidity
Source: PLoS One. 2024 Jul 11;19(7):e0305462. doi: 10.1371/journal.pone.0305462 (PMC11239070; doi:10.1371/journal.pone.0305462)
Supplement: Attachment — Submitted filename: PONE-D-24-00544_review.docx [file pone.0305462.s003.docx]

This paper examines the habitat occupancy of a threatened and specialized species, the diademed plover, along peatlands in the Vilamas wetland, in High Andes. The study provides valuable data for the conservation of the diademed plover, especially considering the low information available for this species mainly because of its reluctance to human eye and the difficulty to access to their territories. I do not have major concerns about this paper, the methods are suitable and the main text is clear and concise. However, I have some minor concerns (see below) and some questions about methods. Why using the 4 NDVI variables in the models since you did not have any prediction about this? Indeed, you stated in page 9 that NDVI was included in the models but it was not linked to any initial hypothesis and it did not significantly associated to occupancy. I suggest to either directly remove all methods and analyses in relation to NDVI or to explain why you included NDVI in the models (hypothesis and prediction; in Introduction) and why you finally did not include it in ranked models (in Methods).

**Minor comments**

Since there is no track-numbering in the manuscript, I numbered text lines for each page. Comments are done following this numbering.

*Page 2 – Abstract*

Ln 2: Add the scientific name to diademed plover.

Ln 5: “being well-suited to perform occupancy models”.

Ln 6: diademed plovers.

Ln 6: remove may.

Ln 8: diademed plovers.

Ln 12: These results are referred to survey data or model prediction data? If it is referred to survey data, it should be 47%. In this case change to: “Approximately 50% of the studied peatlands…”

Ln 16: Add the meaning of NDWI.

Ln 18: diademed plovers.

Ln 18-20: Maybe join the two results in one sentence, saying that your 2 predictions were not supported by data (namely plover occupancy was positively related to NDWI and negatively related to grazing).

*Page 3*

Ln 6: “Understanding what factors determine species’ spatial distribution is an important question in population ecology”.

Ln 7: “knowing where a species…”

Ln 12: “Patches may be unoccupied for a number of reasons. For example, due to their abiotic conditions or the presence/absence of other species (1,2)”.

*Page 4*

Ln 5: “constituting oases within…”

Ln 5: remove “not surprisingly”.

Ln 8: “However, Andean peatlands are now facing rapid changes driven primarily by regional aridization (16-18) and mining activities (19-21)”.

Ln 10: add the species name of the invasive trout.

Ln 11: “To address the consequences of these threats, we need…”

Ln 17: 4,000 m above sea level (m a.s.l.).

*Page 5*

Ln 5: remove “if any”.

Ln 13: add coma after random effects.

Ln 17: “are hypothetised to harbour…”

*Page 6*

Ln 3: diademed plover.

Ln 4: add the scientific name of vicuñas (*Lama vicugna*). Also, scientific name of llama is incorrectly written (*Lama glama*).

Ln 7: “The aim of this paper…”

Ln 14: “to ask whether they…”

Ln 17: plovers instead of birds.

*Page 7*

Ln 5: Do not mention here that you performed surveys, since they are described later in methods. Instead, you should start describing the study are (e.g. The study region is located at Lagunas de Villama…).

Ln 9: “It was declared Ramsar site in 2000, and Important Bird Area (IBA) in … (add year)”.

Ln 12: Maybe “arid cold desert”.

Ln 12: “an extreme temperature amplitude”.

Ln 14: “ranging from 100 to 300 mm per year. There are often…”

*Page 8*

Ln 3: Maybe explain briefly types 1 and 2 to understand better the peatland ecosystem.

Ln 5: Also, maybe explain briefly the Kolla (or Qulla) indigenous community; i.e. from where they come, their transhumance, etc. In this sense, non-native people could understand better the Andean peatland system.

Figure 1: Add the scale in the two maps and the 4 cardinal directions.

Ln 10: “and females lay 2 eggs…”

Ln 13: provision or feed instead of raise.

Ln 15: plovers instead of they.

*Page 9*

Ln 4: “using the Google Earth Engine (GEE) platform”.

Ln 6: topographic position index (TPI).

Ln 9: use only TPI, as you described it before.

Ln 11: “The spectral variables include…” Also, remove all methods concerning NDVI (see my general comments).

Ln 13: add the resolution in pixels of Lansat-8 images.

Ln 14: explain how NDWI is calculated from Landast-8 bands. NDWI = (B3-B4)/(B3+B4).

Ln 15: “We used NDWI as proxy to test our predictions…”

*Page 10*

Ln 9: researchers instead of people.

Ln 14: add the ID of permits/approval (here or in an ethic statement at the end of the manuscript).

Ln 15: “The grazing pressure was estimated as the number of…”

Ln 16: Did you note the presence of llamas/vicuñas in the afternoon while doing surveys? You mention that surveys were done until 17:00h. If no llama/vicuña was detected along the day, you should mention it.

Ln 17: better not to refer to sheep equivalents as SE (because it can be confused with standard error). Simply refer as sheep equivalents.

*Page 11*

Ln 6: add coma after observer.

Ln 12: add the R version

Ln 15: Number the 14 variables (8 statistics NDWI, NDVI, 5 spatial, grazing).

Ln 16: Why is the reasoning behind the selection of the 4 variables selected?

*Page 12*

Ln 5: reference the Appendix 1 when you mention the 24 fitted models.

Ln 8: This sentence is a bit confusing, please, re-write.

Ln 11: How covariates were standardized? Scaling? Describe it briefly.

*Page 13*

General comment in results: why you do not present the data relative to grazing of llamas and vicuñas (sheep-equivalents)? I suggest to add descriptive statistics in results, as you mention later in the discussion some of the results.

Ln 4: diademed plovers.

Ln 4: 18 out of 40.

Ln 5: 13 out of 40.

Ln 6-9: move this part to methods and report here only the results relative to figure 2.

Ln 15: Photograph instead of view. Also, it would be better if the margins of the plover photo match with the margins of the peatland photo.

Ln 18: “both included NDWI as a covariate affecting occupancy”.

*Page 14*

Ln 8: “and was also slightly better than the best ranked model (Table 1)”.

Ln 11-14: You can remove this part and shorten saying that models including grazing as covariate did not perform better than the best ranked model (see appendix 1).

*Page 15*

Table 1: Add a new row in the top to separate the two non-spatial models and the spatial model.

Ln 1: “95% confidence intervals (in parentheses) of three models…”

Ln 2: “The table shows results…”

Ln 6: indicate that 0.58 is the estimate and 0.32-0.79 is the 95%-CI.

Ln 7: indicating instead of suggesting.

Ln 10: remove “i.e. humidity”. Also change to: “(Table 1; Fig. 3)”.

*Page 16*

Figure 3: Better refer to Fig3a and Fig3b instead of left and right panel. Also remove the correlation in the figure legends as it is posted in the main text.

Line 10: 33% instead of 32% (to report the same % as in results).

Ln 11: “chose locations in their upper portions, near the headwaters”.

Ln 12: number the two predictions: “We predicted that: (1) occupancy would increase…; and (2) occupancy would decline…”

Ln 15: “unoccupied peatlands in their abiotic conditions, supporting…”

Ln 16: two predictions instead of all predictions.

*Page 17*

Ln 4-8: In this part of the discussion, you state that drier peatlands should support a lower food availability for plovers than more humid peatlands. However, neither in the introduction nor here you show a clear relationship between these two variables. The NDWI is a surrogate of humidity, but maybe it is not related to food abundance and/or availability in your study system. I would add that more studies are needed to examine the food resources for plovers in weatlands differing in humidity (or NDWI), so your results are maybe not “unexpected”.

Ln 19: separate into two sentences.

*Page 18*

Ln 1: Your ongoing work (58) examines the within-peatland migration or altitudinal migration in the Andes? If the first option is correct, I understand why plovers choose headwaters instead of lower parts of peatlands.

I think references 58 and 59 are incorrectly numbered (58 should be 59 and viceversa).

Ln 4: add scientific name (*Charadrius nivosus*).

Ln 5: “with nests being initiated at those times…”

Ln 7: start a new sentence after the reference 60.

Ln 8: “nest flooding in several coastal species (61)”.

Ln 12: should examine.

Ln 16: separate into two sentences: “food availability. Diademed plovers…”

Ln 17: Two observations would support this hypothesis”.

*Page 19*

Ln 1: “include” is duplicated.

Ln 3: should examine. Also, start this new paragraph directly with the grazing.

Ln 5: Two reasons could explain the lack…”

Ln 5: I do not see how the first reasoning can invalidate your findings, since llamas forage in the same period as plovers reproduce (December-February). One could expect some competitive exclusion by llamas, as they would pressure plover nests and/or territories.

Ln 8: sheep-equivalents instead of se.

Ln 8: in relation to the two reasonings, the lack of effect could be attributable to the fact that llamas and vicuñas were counted only in the morning, when starting the surveys. Maybe if you surveyed them during all day, grazing could affect plover occupancy. See my comment above.

Ln 10: add the scientific names of dunlins and snipes.

Ln 13: even if llamas exert a slight threat to nest success, this…”

Ln 15: “pastoral way of life (…), may be essential for Diademed plover conservation in the mid-term”.

Ln 20: remove the colon.

*Page 20*

Ln 11: “Vilama peatlands are among the highest proportion of occupied peatlands across the species range”.

Ln 15: “climate variability, including the El Niño-Southern Oscillation (ENSO) which strongly affects NW Argentina”.

Ln 19: “affect bird’s occupancy and demography”.
