# Supplementary material for: Habitat occupancy of the threatened Diademed Plover (Phegornis mitchellii) is not affected by llama grazing or peatland size, but declines with peatland humidity
Source: PLoS One. 2024 Jul 11;19(7):e0305462. doi: 10.1371/journal.pone.0305462 (PMC11239070; doi:10.1371/journal.pone.0305462)
Supplement: Attachment — Submitted filename: Response_to_reviewers_PLOS .docx [file pone.0305462.s004.docx]

Dear Dr. Ruiz-Rodriguez,

We are submitting a revised version of our manuscript "**Habitat occupancy of the threatened Diademed Plover (*Phegornis mitchelli*) is not affected by llama grazing or peatland size, but declines with peatland humidity" for your consideration as a research article** in PLOS ONE. We are grateful to you and to the reviewers for your suggestions. The comments of the reviewers were very valuable and they included many good suggestions that we hope have been addressed.

A few of the reviewers’ comments regarded the English. The manuscript has been thoroughly revised by two coauthors who are native English speakers and we hope it is both attractive and understandable to all readers.

Both reviewers asked us to tone down a few sentences that relate peatland size and humidity to food availability, in the Introduction and Discussion. We removed the corresponding sentences from the Discussion and toned down the sentence in the Introduction.

In addition, as suggested by the editorial team, we checked that our manuscript adheres to PLOS ONE guidelines. We have removed our funding from the Acknowledgements section. We have added an ethics statement and permit numbers in the Methods section.

Below, we present detailed responses (in bold) to all of your and the reviewers’ suggestions.

Sincerely,

Alejandro Pietrek, on behalf of all authors.

**#Reviewer 1**

This paper examines the habitat occupancy of a threatened and specialized species, the diademed plover, along peatlands in the Vilamas wetland, in High Andes. The study provides valuable data for the conservation of the diademed plover, especially considering the low information available for this species mainly because of its reluctance to human eye and the difficulty to access to their territories. I do not have major concerns about this paper, the methods are suitable and the main text is clear and concise. However, I have some minor concerns (see below) and some questions about methods. Why using the 4 NDVI variables in the models since you did not have any prediction about this? Indeed, you stated in page 9 that NDVI was included in the models but it was not linked to any initial hypothesis and it did not significantly associated to occupancy. I suggest to either directly remove all methods and analyses in relation to NDVI or to explain why you included NDVI in the models (hypothesis and prediction; in Introduction) and why you finally did not include it in ranked models (in Methods).

**We have removed references to NDVI throughout the text. We did not incorporate NDVI among the variables of the occupancy models. We did visually explore Diademed Plover occurrence as a function of NDVI descriptive statistics, but we have removed this from the paper to focus only on the variables that we did include in occupancy models.**

**Minor comments**

Since there is no track-numbering in the manuscript, I numbered text lines for each page. Comments are done following this numbering.

*Page 2 – Abstract*

Ln 2: Add the scientific name to diademed plover.

**R: We added the scientific name. (Line 4)**

Ln 5: “being well-suited to perform occupancy models”.

**R: We changed the sentence to read "Andean peatlands are well-suited to occupancy modelling because they are discrete patches of humid habitat within a matrix of high-altitude steppe”. Lines (Lines 5-7)**

Ln 6: diademed plovers.

**R: We rewrote this as Diademed Plovers. (Line 7)**

Ln 6: remove may.

**R: We removed “may”. (Line 7)**

Ln 8: diademed plovers.

**R: We rewrote this as Diademed Plovers. (Line 9)**

Ln 12: These results are referred to survey data or model prediction data? If it is referred to survey data, it should be 47%. In this case change to: “Approximately 50% of the studied peatlands…”

**R: We changed the sentence to read “Occupancy models showed that more than 50 % of the studied peatlands were used by Diademed Plovers”, to clarify that the statement refers to model predictions. (Lines 14-15).**

Ln 16: Add the meaning of NDWI.

**R: We clarified the meaning of NDWI: "Normalized Difference Water Index, an index correlated with water content and humidity". (Lines 18-19)**

Ln 18: diademed plovers.

**R: Changed to Diademed Plovers. (Line 20)**

Ln 18-20: Maybe join the two results in one sentence, saying that your 2 predictions were not supported by data (namely plover occupancy was positively related to NDWI and negatively related to grazing).

**R: No change made. We considered the suggestion but found the text clearer when we kept these as two concise and shorter sentences.**

*Page 3*

Ln 6: “Understanding what factors determine species’ spatial distribution is an important question in population ecology”.

**R: We changed the sentence to read " A major aim of population ecology is to identify the factors that determine how species are distributed in space”. (Lines 32-33)**

Ln 7: “knowing where a species…”

**R: We replaced “understanding” with “knowing” following the reviewer’s advice. (Line 33)**

Ln 12: “Patches may be unoccupied for a number of reasons. For example, due to their abiotic conditions or the presence/absence of other species (1,2)”.

**R: We changed the structure of the paragraph to indicate the three main reasons: First,.... Second,..... Third… (Line 38)**

*Page 4*

Ln 5: “constituting oases within…”

**R: We changed the section to read: "** **Peatlands in the High Andes of South America occur as discrete, humid habitat patches along streams within an arid matrix of high-altitude steppe. These peatlands are oases that harbor highly distinct biological communities, including many endemic amphibians and birds** **(10–12)."(Lines 48-49)**

Ln 5: remove “not surprisingly”.

**R: We removed "not surprisingly."(Line 48)**

Ln 8: “However, Andean peatlands are now facing rapid changes driven primarily by regional aridization (16-18) and mining activities (19-21)”.

**R: We changed the sentence to read: " However, these biodiversity islands are experiencing rapid changes in vegetation related to declining humidity, caused by aridization** **of the climate at a regional scale (16–18) and escalating water requirements for mining activities** **at local scales (19–21). "(Lines 50-53)**

Ln 10: add the species name of the invasive trout.

**R: Added. (Line 54)**

Ln 11: “To address the consequences of these threats, we need…”

**R: We have now re-written the sentence following the reviewer suggestion. (Line 55)**

Ln 17: 4,000 m above sea level (m a.s.l.).

**R: Added (Line 61)**

*Page 5*

Ln 5: remove “if any”.

**R: We removed "if any". (Line 71)**

Ln 13: add coma after random effects.

**R: Added. (Line 80)**

Ln 17: “are hypothetised to harbour…”

**R: No change made. We are linking the formal theory and evidence on patch size/population size to peatlands and describing where our system might fit. There are no formal hypotheses on peatland size effects on occupancy. (Line 86)**

*Page 6*

Ln 3: diademed plover.

**R: We rewrote as Diademed Plovers. (Line 108)**

Ln 4: add the scientific name of vicuñas (*Lama vicugna*). Also, scientific name of llama is incorrectly written (*Lama glama*).

**R: We have corrected the scientific name of lamas and added the scientific name for vicuñas. According to the red list published by the Argentinean Society of Mammalogists the scientific name of vicuñas is still *Vicugna vicugna*.**(Arzamendia, Y.; Acebes, P.; Baldo, J. .; Rojo, V. ; Segovia, J.M. 2019) **(Lines 93-96)**

Ln 7: “The aim of this paper…”

**R: We rewrote the sentence following the suggestion of the reviewer. (line 101)**

Ln 14: “to ask whether they…”

**R: We changed the sentence to read " Additionally, we explored the locations, within peatlands, used by the Diademed Plovers, to determine whether plovers were distributed randomly or toward one end of these somewhat linear habitats." (Lines 107-109)**

Ln 17: plovers instead of birds.

**R: We replaced “birds” with “plovers”. (Line 111)**

*Page 7*

Ln 5: Do not mention here that you performed surveys, since they are described later in methods. Instead, you should start describing the study are (e.g. The study region is located at Lagunas de Villama…).

**R: We have now changed the sentence and it now reads “We conducted our study at…” (Line 120)**

Ln 9: “It was declared Ramsar site in 2000, and Important Bird Area (IBA) in … (add year)”.

R: **Added (Line 124)**

Ln 12: Maybe “arid cold desert”.

**R: We have followed here the description of Peel et al. 2007 that classifies the climate type as “arid desert cold”.**

Ln 12: “an extreme temperature amplitude”.

**R: Done. (Line 127)**

Ln 14: “ranging from 100 to 300 mm per year. There are often…”

**R: We have now split this in two sentences as suggested by the reviewer. (Line 129)**

*Page 8*

Ln 3: Maybe explain briefly types 1 and 2 to understand better the peatland ecosystem.

**R: We have now specified the two cushion plants that characterize these peatlands (*Distichia* and *Oxychloe*), and further detail they occur in relatively steep areas above 4,000 m . (Lines 136-138)**

Ln 5: Also, maybe explain briefly the Kolla (or Qulla) indigenous community; i.e. from where they come, their transhumance, etc. In this sense, non-native people could understand better the Andean peatland system.

**R: We have now added one sentence to describe general details on the Kollas people. The sentence now reads: “Each summer between late November and early February, Kolla shepherds from the neighboring community of Lagunillas del Farallón move up to Vilama to graze their llamas on the peatlands, but no people live at Vilama year-round. The Kollas comprise a group of tribes that inhabit northwest Argentina, northern Chile, and Bolivia and have held common land property in this area for centuries”. (Lines 141-143)**

Figure 1: Add the scale in the two maps and the 4 cardinal directions.

**R: We added the scale and north arrow.**

Ln 10: “and females lay 2 eggs**…”**

**R: We added "females" to the original sentence. (Lines 64-65)**

Ln 13: provision or feed instead of raise.

**R: We changed it to 'provision'. (Line 66)**

Ln 15: plovers instead of they.

**R: We replaced “they” with “plovers” (Line 68)**

*Page 9*

Ln 4: “using the Google Earth Engine (GEE) platform”.

**R: Corrected. (Line 155)**

Ln 6: topographic position index (TPI).

**R: Corrected. (Line 156)**

Ln 9: use only TPI, as you described it before.

**R: Corrected. (Line 159)**

Ln 11: “The spectral variables include…” Also, remove all methods concerning NDVI (see my general comments).

**R: We removed the sentences about NDVI throughout the text because we did not incorporate NDVI statistics in the occupancy models.**

Ln 13: add the resolution in pixels of Lansat-8 images.

**R: We added the resolution of the Landsat imagery. (Line 165)**

Ln 14: explain how NDWI is calculated from Landast-8 bands. NDWI = (B3-B4)/(B3+B4).

**R: We have now added the NDWI equation in the methods (Lines 158-160 of the revised version). The sentence now reads: “NDWI is a widely used index to assess water content in plants and areas prone to flooding or waterlogging** **(47–49) and is calculated using the green and near infrared band as** $\left( \boldsymbol{G-NIR} \right)\boldsymbol{/(G+NIR)}$**”. (Lines 161-163)**

Ln 15: “We used NDWI as proxy to test our predictions…”

**R: Corrected. (Line 166)**

*Page 10*

Ln 9: researchers instead of people.

**R: We now use researchers instead of people. (Line 177)**

Ln 14: add the ID of permits/approval (here or in an ethic statement at the end of the manuscript).

**R: We have now added the permit numbers here and the ethics statement. (Lines 185-188)**

Ln 15: “The grazing pressure was estimated as the number of…”

**R: We changed the phrase to "Grazing pressure was estimated from the number of..." (Line 189)**

Ln 16: Did you note the presence of llamas/vicuñas in the afternoon while doing surveys? You mention that surveys were done until 17:00h. If no llama/vicuña was detected along the day, you should mention it.

**R: We added the following sentence: "We detected both llamas and vicuñas in the morning and afternoon, and we did not notice a temporal pattern in the use of peatlands by these camelids." (Lines 194-195)**

Ln 17: better not to refer to sheep equivalents as SE (because it can be confused with standard error). Simply refer as sheep equivalents.

**R: We changed to "sheep equivalents" throughout the manuscript.**

*Page 11*

Ln 6: add coma after observer.

**R: Added. (Line 201)**

Ln 12: add the R version

**R: Added. (Line 207)**

Ln 15: Number the 14 variables (8 statistics NDWI, NDVI, 5 spatial, grazing).

**R: We clarified what the ten (after removing NDVI statistics) variables are. (Line 211)**

Ln 16: Why is the reasoning behind the selection of the 4 variables selected?

**R: We have justified the use of area and grazing in the introduction. However we agree that the use of mean NDWI as the chosesn statistic merits further explanation. In lines 216-217 we wrote:** “**NDWI min (the minimum NDWI min recorded over three years and the range of NDWI ( max NDWI- min NDWI) were both correlated with NDWI mean, but we decided to use NDWI mean as a more reliable statistics of the long term moisture dynamics of peatlands.” (Lines 216-218)**

**As for slope in lines 213-214 we wrote: “Slope was the only variable for which we did not have specific predictions and we included it in a more exploratory way”.**

*Page 12*

Ln 5: reference the Appendix 1 when you mention the 24 fitted models.

R: **Reference added. (Line 223)**

Ln 8: This sentence is a bit confusing, please, re-write.

R: **We rewrote the section as follows: "Twelve of the models assumed detection was constant. Each of these twelve models included one or two of the following as covariates of occupancy: peatland area, mean NDWI, grazing, and slope. One model included, as an occupancy covariate, the interaction between peatland area and grazing. The remaining twelve models were basically the same except that instead of assuming detection was constant, they included peatland area as a detection covariate”. (Lines 225-229)**

Ln 11: How covariates were standardized? Scaling? Describe it briefly.

**R: The sentence now reads: “****All covariates were standardized for statistical modeling using the function scale () in R.” (Line 229-230)**

*Page 13*

General comment in results: why you do not present the data relative to grazing of llamas and vicuñas (sheep-equivalents)? I suggest to add descriptive statistics in results, as you mention later in the discussion some of the results.

**R: We added median values and range of the Grazing variable. (Lines 266-267)**

Ln 4: diademed plovers.

**R: Added. (Line 242)**

Ln 4: 18 out of 40.

**R: No change made. "18 of 40" is grammatically correct and more concise. (Line 242)**

Ln 5: 13 out of 40.

**R: No change made. "13 of 40" is also correct and more concise. (Line 243)**

Ln 6-9: move this part to methods and report here only the results relative to figure 2.

**R: We moved the sentence "** **We used Google Earth to measure the Euclidean distance (horizontal) from the top of the peatland (headwaters) to the nest (if found) or the average location where plovers were recorded" to lines 183-184 under methods.**

Ln 15: Photograph instead of view. Also, it would be better if the margins of the plover photo match with the margins of the peatland photo.

**R: We changed “View” to “Photograph”. The margins of the plover photo now fit the peatland photo (Line 251)**

Ln 18: “both included NDWI as a covariate affecting occupancy”.

**R: Changed. (Line 254)**

*Page 14*

Ln 8: “and was also slightly better than the best ranked model (Table 1)”.

**R: We changed the phrase to "and was also slightly better than the best ranked model (Table 1)". (Line 261)**

Ln 11-14: You can remove this part and shorten saying that models including grazing as covariate did not perform better than the best ranked model (see appendix 1).

**R: We rewrote the sentence: "Models that included grazing as a covariate (median grazing: 1.78 sheep equivalents, range: 0-9.79 sheep equivalents) did not perform better than the best ranked non-spatial model (Appendix I).". (Lines 265-267)**

*Page 15*

Table 1: Add a new row in the top to separate the two non-spatial models and the spatial model.

**R: Added. (Table 1)**

Ln 1: “95% confidence intervals (in parentheses) of three models…”

**R: Added. (Caption Table 1)**

Ln 2: “The table shows results…”

**R: Changed. (Caption Table 1)**

Ln 6: indicate that 0.58 is the estimate and 0.32-0.79 is the 95%-CI.

**R: Added. (Line 272)**

Ln 7: indicating instead of suggesting.

**R: Changed. (Line 272)**

Ln 10: remove “i.e. humidity”. Also change to: “(Table 1; Fig. 3)”.

**R: Removed. (Line 276)**

*Page 16*

Figure 3: Better refer to Fig3a and Fig3b instead of left and right panel. Also remove the correlation in the figure legends as it is posted in the main text.

**R: Correlation removed from the figure legend. As the left panel comprises two plots, we prefer to leave the reference as in the original version. (Caption Figure 3)**

Line 10: 33% instead of 32% (to report the % as in results).

**R: Corrected. Thank you for the observation. (Line 286)**

Ln 11: “chose locations in their upper portions, near the headwaters”.

**R: We rewrote the sentence: "Plovers did not establish their breeding territories randomly within peatlands, but instead chose locations in the upper portions, near the headwaters." (Lines 286-287)**

Ln 12: number the two predictions: “We predicted that: (1) occupancy would increase…; and (2) occupancy would decline…”

R: **Added. (Lines 287-291)**

Ln 15: “unoccupied peatlands in their abiotic conditions, supporting…”

**R: We changed the sentence to read " Occupied peatlands differed in humidity from unoccupied peatlands". (Line 291)**

Ln 16: two predictions instead of all predictions.

**R: We rewrote these as “contrary to our predictions”. Within the sentence, we more broadly discuss slope in addition to peatland size, humidity and grazing. (Line 292)**

*Page 17*

Ln 4-8: In this part of the discussion, you state that drier peatlands should support a lower food availability for plovers than more humid peatlands. However, neither in the introduction nor here you show a clear relationship between these two variables. The NDWI is a surrogate of humidity, but maybe it is not related to food abundance and/or availability in your study system. I would add that more studies are needed to examine the food resources for plovers in wetlands differing in humidity (or NDWI), so your results are maybe not “unexpected”.

**R: We removed the first sentence: “Contrary to the expectation that plovers would select more humid peatlands to maximize food availability, plovers instead selected less water-saturated peatlands”. The first sentence now reads:** “**Diademed Plovers selected less water-saturated peatlands” (Line 297). We added the point that further studies are needed to explore the relation between peatland humidity and productivity/diversity of macroinvertebrates in the Andes in line 91 of the revised introduction. After an extensive literature search, we agree with the reviewer that there is no clear link between Andean peatlands humidity and macroinvertebrate productivity/diversity.**

Ln 19: separate into two sentences.

**R: We split it into two sentences: "** **Although Diademed Plovers may occasionally stay year-round at Vilama, the peatlands they use during the breeding season can be frozen for weeks or months. Ongoing work tracking Diademed Plovers among peatlands (58) suggests that plovers often migrate to lower altitudes. ".**

*Page 18*

Ln 1: Your ongoing work (58) examines the within-peatland migration or altitudinal migration in the Andes? If the first option is correct, I understand why plovers choose headwaters instead of lower parts of peatlands.

**R: We changed the sentences to: "Although Diademed Plovers may occasionally stay year-round at Vilama, the peatlands they use during the breeding season can be frozen for weeks or months. Ongoing work** **tracking Diademed Plovers among peatlands (58) suggests that plovers often migrate to lower altitudes. By selecting less-saturated peatlands at the onset of the breeding season, and nesting near the headwaters, Diademed Plovers might reduce their risk of nest flooding as water availability increases in spring."**

I think references 58 and 59 are incorrectly numbered (58 should be 59 and viceversa).

**Our ongoing work follows the larger scale altitudinal migration that occurs with plovers (between peatlands). References were correctly numbered, as 58 was cited in the text before 59.**

Ln 4: add scientific name (*Charadrius nivosus*).

**R: Added. (Line 315)**

Ln 5: “with nests being initiated at those times…”

**R: No changes made. The sentence is grammatically correct. (Line 317)**

Ln 7: start a new sentence after the reference 60.

**R: Changed. (Line 319)**

Ln 8: “nest flooding in several coastal species (61)”.

**R: Changed. (Line 320)**

Ln 12: should examine.

**R: Changed. (Line 321)**

Ln 16: separate into two sentences: “food availability. Diademed plovers…”

**R: We rewrote the sentence and deleted the reference to food availability. It reads:** **“Although we predicted that larger peatlands would be more likely to be occupied by plovers, Diademed plovers were often recorded in relatively small peatlands. Diademed Plovers may require only a small foraging area to meet their food requirements” (Lines 324-326)**

Ln 17: Two observations would support this hypothesis”.

**R: We kept the phrase as: "Two observations suggest that Diademed Plovers have small home ranges". We prefer not to raise and discuss new hypotheses in the Discussion section, and we find it clearer to refer specifically to the idea that they have small home ranges, given that there are several ideas expressed in the previous sentence. (Lines 326-327).**

*Page 19*

Ln 1: “include” is duplicated.

**R: Removed. (Line 331)**

Ln 3: should examine. Also, start this new paragraph directly with the grazing.

**R: Changed. (Line 334)**

Ln 5: Two reasons could explain the lack…”

**R: Changed. (Line 334-335)**

Ln 5: I do not see how the first reasoning can invalidate your findings, since llamas forage in the same period as plovers reproduce (December-February). One could expect some competitive exclusion by llamas, as they would pressure plover nests and/or territories.

**R: We added: “Thus, effects of overgrazing and more generally peatland degradation are restricted to only three months per year”. (Lines 336-337)**

Ln 8: sheep-equivalents instead of se.

**R: Changed. (Line 338)**

Ln 8: in relation to the two reasonings, the lack of effect could be attributable to the fact that llamas and vicuñas were counted only in the morning, when starting the surveys. Maybe if you surveyed them during all day, grazing could affect plover occupancy. See my comment above.

**R: We added: "** **Depending of size and location, each survey could last up to 2 hours, and as many as 5-6 peatlands were visited in the same day" (lines 178-179 under methods). Llamas and vicuñas were recorded at the beginning of each *survey* and not at the beginning of the day (lines 189-190). We added " We detected llamas and vicuñas in the morning and afternoon, and we did not notice a temporal pattern in the use of peatlands by either of these camelids." (Lines 194-195).**

Ln 10: add the scientific names of dunlins and snipes.

**R: Added. (Lines 340-341)**

Ln 13: even if llamas exert a slight threat to nest success, this…”

**R: We rewrote as: “suggesting that even if llamas were a slight threat to nest success, this threat would not be enough to impact the population viability”. Lines 344-345)**

Ln 15: “pastoral way of life (…), may be essential for Diademed plover conservation in the mid-term”.

**R: We changed the sentence to: " Considering that the Indigenous pastoral way of life maintains peatland habitat (in the face of mining interests, for example), preserving this way of life may be essential to the goals of plover conservation in the medium term."(Lines 345-347)**

Ln 20: remove the colon.

**R: Changed to a comma. (Line 350)**

*Page 20*

Ln 11: “Vilama peatlands are among the highest proportion of occupied peatlands across the species range”.

**R: We changed the text to read: " Efforts are underway to assess global populations of Diademed Plovers (Manomet inc. in preparation). They confirm that the proportion of occupied peatlands at Vilama wetlands is among the highest range-wide, highlighting the importance of Vilama for this globally near-threatened species." (Lines 362-365)**

Ln 15: “climate variability, including the El Niño-Southern Oscillation (ENSO) which strongly affects NW Argentina”.

**R: We followed the suggestion of the reviewer and expanded the meaning of the acronym. (Line 367)**

Ln 19: “affect bird’s occupancy and demography”.

**R: We changed the text to: “avian occupancy and demographics". (Lines 371-372)**

**Reviewer 2**

The goal of this paper is to identify peatland features that Diademed Plovers use during their breeding season when they establish delimited territories within peatlands. This study is intriguing, particularly for a species with limited information on its biology and ecology. I commend the authors for the extensive sampling effort undertaken for this research. I believe that the manuscript is publishable, but from my point of view, it could benefit from restructuring to organize ideas and enhance readability. Also, I am not a native English speaker, but I believe it requires an English formatting review.
Some examples I suggest are:
Introduction:
1- Rewrite the hypotheses paragraph, being more concise: "The hypotheses in this work are… The predictions are…"

**R: We re-wrote as two sentences, the first on hypothesis and the second on predictions. “Our hypotheses are that plovers would respond positively to peatland size and negatively to grazing by llamas and vicuñas. Thus, we predict that plover occupancy would increase with peatland size and humidity and decline with grazing pressure.” (Lines 104-107).**

2- Are there no other predation pressures on these sites for plovers aside from grazing?

**R: We are here considering grazing a potential threat to nests but not actual predation. Aplomado falcons, culpeo foxes and wild cats can potentially prey on adults and chicks but we do not believe is necessarily relevant to our study.**

3- On page 5, the sentence: “Importantly, their specific micro-habitat requirements remain largely unknown, and Diademed Plovers are often absent from peatlands that, to the human eye, seem identical to those where individuals are present (28)” It is not clear what is meant.

**R: We rewrote as two sentences for clarity: " Importantly, their specific micro-habitat requirements remain largely unknown. Peatlands used by Diademed Plovers look, to the human eye, very similar to peatlands where they are not found** **(28)." (Lines 69-70)**

4- I recommend omitting the word “rare” when referring to the species, as it may confuse due to its conservation status, in the introduction section.

**R: We removed the word rare from the introduction.**

5- The species could be further characterized, by detailing their wintering and breeding areas. It would be beneficial to specify at which stage of the life cycle they inhabit these areas. Exploring their cohabitation with other species, potential competition, interrelations with other birds and/or animals, as well as the impact of grazing, would also be valuable.

**R: We moved a description of the species from Methods to the Introduction (Lines 64-68, see comment 3 in methods).**

Methods:

1- Sentence: “Peatlands at Vilama can be classified as types 1 and 2 (44): they are generally dominated by plant communities adapted to high altitudes and extreme conditions and located in relatively steep areas” What would be types 1 and 2?

**R: We have added further details on the species that dominate these peatlands and other general features as reviewer 1 also suggested (Lines 136-138 of the revised version). We cite Izquierdo (2022) for further references on these types of peatlands.**
2- Sentence: “Each summer between late November and early February, Kolla shepherds from the neighboring community of Lagunillas del Farallón move up to Vilama to graze their llamas on the peatlands, but no people live at Vilama year- round.” I assume these are the same months plovers occupy the breeding area, but that should be clear.

**R: We changed the sentence to: “Each summer between late November and early February (when most plovers nest), Kolla shepherds from the neighboring community of Lagunillas del Farallón move up to Vilama to graze their llamas on the peatlands. No people live at Vilama in winter”. Lines 138-41 of the revised version.**

3- Sentence: “Diademed Plovers make a rudimentary nest on the ground and lay two eggs in late spring. Diademed Plovers both forage and nest within the stream habitats; nests are placed on the cushion plants or among rocks in rocky patches. Both parents incubate eggs and raise the chicks, which are precocial. The breeding grounds of Diademed Plovers are often inaccessible to observers during the winter, and winter distribution data are scarce. In areas like Vilama, where many peatlands freeze in winter, they might be altitudinal migrants.” This information might be better moved to the introduction for a clearer understanding of the species' ecology.

**R: We moved this section to the Introduction.**

Discussion:

1- Sentence: “During the 2021–2022 breeding season, Diademed Plovers occupied about half of the peatlands at Vilama”. This would be half of the peatlands selected by the authors, right?

**Strictly speaking that is the predicted occupancy on the patches we surveyed, but given peatlands were selected randomly, we infer that can approximate overall occupancy at Vilama. Statistically speaking, we used a sample of peatlands to infer mean occupancy of peatlands at Vilama. We re-wrote the sentence as: “We found surprisingly high occupancy rates; according to the best-ranked model, 50% of the peatlands above 0.4 hectares at Vilama were utilized by Diademed Plovers”. (Lines 350-352)**

2- Sentence: “Although we predicted that peatland size would increase food availability to Diademed Plovers, we did not measure food availability, and Diademed Plovers may require only a small foraging area to meet their food requirements”. I recommend refraining from making predictions in this case, as without analyzing food availability, predicting outcomes in a changing environment is not feasible. While the plover may need relatively small foraging areas, it is possible that during the sampling period, prey availability was insufficient, or specific prey types or sizes were lacking. Without trophic studies, it may be best to avoid speculating on hypotheses, particularly for a species with limited knowledge and difficult access to research. It would be beneficial to note observations for future studies, without proposing specific hypotheses.

**R: We have addressed the reviewer comment in two ways. First, we have removed the references to food availability as we concur with the reviewer we did not measure food availability. The sentence now reads: “Although we predicted that larger peatlands would be more likely to be occupied by plovers, Diademed plovers were often recorded in relatively small peatlands” (Lines 324-325)**

**Second we now more explicitly acknowledge (as reviewer 1 suggested) that further studies linking macroinvertebrate productivity (food) to peatland humidity are needed to really understand the mechanisms behind the patterns we observe. (Line 91 in the introduction)**

3- Sentence: “Grazing pressure was also absent from the most competitive peatland occupancy models. Two reasons stand out to explain the lack of effects of grazing on plover occupancy. First, llamas are not permanent at Vilama and are present only between late November and early February. Second, densities were overall low (2.84 se/ha ± 3) and highly variable, as llamas move freely among peatlands (the number of llamas on a peatland varied among surveys at the same peatland)”. However, they are present and moving randomly precisely during the breeding months, leading the authors to formulate a hypothesis with their prediction. Perhaps this occurrence was coincidental, or the llamas are in wetter peatlands, or the number of llamas grazing is insignificant for the plovers.

This is the proposal put forward by the authors later: “From a conservation perspective, although we did not examine nest survival, Diademed Plovers have coexisted with short-term Indigenous pastoralism at Vilama for thousands of years, suggesting that even if were llamas are a slight threat to nest success, this threat would not be enough to impact the population viability. Moreover, preserving the Indigenous pastoral way of life (in the face of mining interests, for example), and the resultant habitat preservation this maintains, may be essential to the goals of plover conservation in the medium term. Diademed Plovers selected drier peatlands in the breeding season of 2021–2022, but selection patterns may vary temporally in the face of climate variability that includes the ENSO that strongly affects NW Argentina (18,66). The 2021–2022 breeding season was among the wettest in the previous decade. In drier years, small peatlands may become unsuitable (no running water) and peatlands that were too humid for Diademed Plovers in 2021–2022 (large NDWI values) may become suitable. Given that climate may frequently interact with local factors to affect occupancy and demographics of birds (67), it is important to follow up one-season occupancy surveys by replicating occupancy surveys over time, under contrasting conditions, to disentangle the impacts of climate on habitat selection by peatland- and other wetland specialist birds. More generally, understanding interactions between climate and habitat will be critical to understand the effects of climate change on bird populations in the High Andes.”
This is crucial. I recommend beginning with these statements to form hypotheses and predictions, as this data does not arise from this study; instead, it should serve as the foundation of this study, just like the previous data on grazing.

**R: Following the reviewer advice, we have moved part of the information on grazing to lines 91-99 of the revised introduction, to better inform our hypothesis. As for climate variability we had no way of knowing 2021-2022 would be an especially wet year. Building a post-hoc hypothesis (or hypothesis after the results) is a practice that has been questioned recurrently in Ecology and Evolution despite being common** (Fraser *et al.* 2018). **Thus, we preferred to leave our hypothesis on humidity as it now stands. However, we do believe (as we highlight in the discussion) that long-term studies may help to better understand the role of climatic variation on Diademed plover occupancy (Particularly if we can have a sample of dry and wet years).**

**Arzamendia, Y.; Acebes, P.; Baldo, J. .; Rojo, V. ; Segovia, J.M.** 2019. Vicugna vicugna | Categorización de los mamíferos de Argentina.

**Fraser, H., Parker, T., Nakagawa, S., Barnett, A. & Fidler, F.** 2018. Questionable research practices in ecology and evolution. *PLOS ONE* **13**: e0200303.
