# Supplementary material for: Habitat occupancy of the threatened Diademed Plover (Phegornis mitchellii) is not affected by llama grazing or peatland size, but declines with peatland humidity
Source: PLoS One. 2024 Jul 11;19(7):e0305462. doi: 10.1371/journal.pone.0305462 (PMC11239070; doi:10.1371/journal.pone.0305462)
Supplement: Attachment — Submitted filename: Response_to_reviewers2.docx [file pone.0305462.s005.docx]

Dear Dr. Rodrigues Paiva,

We are submitting our second revised version of our manuscript "**Habitat occupancy of the threatened Diademed Plover (*Phegornis mitchelli*) is not affected by llama grazing or peatland size, but declines with peatland humidity" for your consideration as a research article** in PLOS ONE. We are grateful to you and to the reviewers for your suggestions. The comments of the reviewers were very valuable and they included many good suggestions that we hope have been addressed.

Below, we present detailed responses (in bold) to all of your and the reviewers’ suggestions.

Sincerely,

Alejandro Pietrek, on behalf of all authors.

**Reviewer 3**: This revised paper provides valuable ecological information for an understudied species in a unique system. The study design and analysis are robust, and the writing is clear. I only have minor suggestions below.

Line 16: Are there other plover species that occupy Vilama and the peatlands are important for their conservation as well? If you are referring to diademed plovers, perhaps change ‘plover conservation’ to ‘the conservation of this species’.

**R: We changed it to “Diademed Plover conservation” to make it more specific.**

Line 20: I do think the last paragraph of the Discussion provides some needed context that the 2021-2022 breeding season was wetter than average years and this could be noted in the Abstract. It felt like a big reveal at the very end of the paper that would have been nice to know up front.

**R: Thanks for the suggestion. We agree with the reviewer and we have now introduced two important changes. In line 20 of the abstract we wrote: “This may be especially important in wet years, like the year when we conducted our surveys”. Additionally, under the Study Area description we added:** “**There are often inter-annual fluctuations in rainfall, alternating between wet and dry periods** **[18]. For example, mean annual precipitation between 2008 and 2019 was 177 mm (range: 65-315), and precipitation between July 2021 and June 2022 was 244 mm (38 % over the annual mean)”** **(Lines 131-133)**

Line 87: Perhaps ‘greater diversity of micro-habitats’ instead of ‘more’

**R: Changed.**

Figure 1: Maybe it’s just the resolution of the image that I am seeing in the document, but the different dot sizes are not very apparent and I’m wondering if size is worth including in the Figure without a legend indicating the magnitude of variation. In the caption, does the color refer to the latent spatial random effect (meaning more plovers in the red dots and less plovers in the blue dots)? I am not sure how to interpret “spatial random error”, but I would suspect you mean to say the value of the spatial effect at each site (positive or negative). It’s also interesting that there seems to be an east to west effect where there were more plovers in the eastern sites and less in the western sites. I’m wondering if the authors can hypothesize why there seems to be some clustering almost split in the middle of the surveyed sites.

**R: We changed Figure 1 so that points do not include spatial random effects and improved our explanation of the legend: “Vilama wetlands. Each dot represents a surveyed peatland”. Because the interpretation of random spatial errors can be tricky for non-occupancy modelers we have decided to move figure 1 to the appendix and replace it with a figure where we only show the spatial location of surveyed peatlands.**

**We appreciate the reviewer’s inquiry about interpreting spatial random errors. These are not latent occupancy states and therefore they do not tell us anything in terms of the likelihood of a patch being occupied. Depending on the values of other covariates in the model, two response values (i.e., the latent occupancy z as well as occupancy probability psi) at sites with similar values of the random effect may or may not be similar. If the covariates have similar values, they will be very similar. If not, they could be different, which depends on the estimated magnitudes of the covariate effects relative to the spatial random effect values.**

**We doubt there is a strong precipitation gradient in only 40 km and peatlands are fed by several mountains, therefore there is not an obvious explanation we feel comfortable speculating on. However, a plot of mean NDWI mean (our main predictor) against longitude in UTM coordinates does show some correlation between NDWI and space (with more peatlands showing higher NDWI values to the west). We believe these spatial pattens we observe (both for the random effects and partially for some of the covariates) support the incorporation of space to the models.**

**Fig 1. Mean NDWI plotted against UTM easting. In blue, peatlands where plovers were detected, in red peatlands where plovers were not detected. The plot shows some correlation between space and NDWI.**

Line 126-130: Related to my previous comment, it would be beneficial to add how precipitation (and maybe temperature) during the study year compared to these general descriptions of climate conditions.

**R: As described in our response to the reviewer's second comment, we added a sentence describing precipitation means and variation over the years before the study (Lines 131-133)**

Line 228: Rather than ‘basically the same’, state that the covariates on occupancy were the same as the 12 models with constant detection probabilities.

**R: Changed.**

Line 230: What coordinates were used for the spatial random effect? Was it the centroid of each peatland, the starting point, or something else? The centroid would seem to be the least affected by the size of the peatland.

**R: This is an important comment and we have now clarified this in lines 235-237. “To estimate random effects in the spatial models we used an exponential covariance function, where UTM coordinates were defined at the centroid of each peatland”**

Line 234: Change ‘make’ to ‘made’

**R: Changed.**

Line 260: Easier to fit in what way?

**R: We usually say a model is easy to fit if it does not require large computational effort and reliably converges to a solution. To make this clear now the sentence reads: “easier to converge.” (line 264)**

**Reviewer 4:  This paper presents new data on factors that affect peatland use by a little-studied species, the Diademed Plover. The study is overall well-executed, and the statistical analyses are appropriate for the study system and research questions. I do not have any major concerns, and the authors have done a commendable job addressing previous reviewer questions. My only general question is to ask the authors how variable was grazing pressure among each peatland? Were there some peatlands that, for example, experienced zero grazing pressure, while others experienced heavy grazing pressure? And are counts of vicunas and llamas during each survey sufficient to characterize the grazing pressure experienced throughout the course of the season? I wonder if an effect could be detected if grazing pressure were measured using different methods, or if there were a wider range of grazing pressures present within the study system.**

**R: 32 out of 40 peatlands were characterized based on at least three visits and, as we wrote in lines 267-269 there was wide variation in grazing pressure (range: 0-9.79 sheep equivalents/ha, median: 1.78). Below we include an exploratory plot of grazing pressure that shows several peatlands have values near zero. In blue, peatlands where plovers where detected, in red peatlands were plovers where not detected.**

**Fig2. Grazing values (sheep equivalents/ha) for the 40 peatlands surveyed.**

**Minor editorial comments given below:**

Abstract
Lines 7-10: These two sentences are somewhat repetitive and can be condensed.

**R: We deleted the second sentence and the previous now reads: “We hypothesized that Diademed Plovers occupy preferably larger and more humid peatlands, and avoid peatlands used for grazing by llamas and vicuñas, which may trample vegetation and nests” (Lines 7-9).**

Introduction
Lines 74-76: This sentence is somewhat awkward and should be restructured.

**R: We re-wrote the sentence and it now reads: “For species like the Diademed Plover, using models based on occurrence (presence or presence-absence) can help identify factors affecting occupancy and also provide a cost-effective method to assess population status based on occupancy rather than abundance”. Lines 74-76.**

Paragraph lines 74-82: This paragraph provides some general discussion of modeling occurrence/occupancy. It could use another sentence or two at the end to explain the relevance of this background information to the current study, perhaps by explaining the appropriateness of the study system for this type of occupancy model.

**R: We added the following sentence before the end of the paragraph: “Site-occupancy models are the preferred option for habitat specialists, especially those not found in all patches and occuring at relatively low densities” (lines 80-81).**

Line 95: coastal habitats?

**R: Added. Thanks for the suggestion.**

Lines 105-107: These two sentences are somewhat repetitive and can be condensed.

**R: This was a single sentence in the original manuscript, but one of the reviewers asked to write separate sentences for hypotheses and predictions. Thus, we are leaving it as it now stands**.

Methods
Line 137: What are these types?

**R: We added the features of these peatlands after the colon, but for further clarification and to more explicitly link this to the work of Izquierdo et al., we re-wrote the sentence as:**

**“Peatlands at Vilama can be classified as types 1 and 2 according to Izquierdo** **et al. [45]: they are generally dominated by cushion plants of the genus *Distichia* and *Oxychloe* and are located in relatively steep areas above 4000 m.a.s.l” (lines139-141).**

Line 155: included

**R: Corrected.**

Line 172: encompassing

**R: No changes made. The sentence is grammatically correct.**

Results
Lines 276-278: The first half of this sentence could go to Methods section, paragraph lines 211-221 where this correlation is discussed. Also, I’m not sure why the plot of detections vs. NDWI min is included in Figure 3, given that this variable was discarded from the occupancy models due to correlation with NDWI mean.

**R: We agree with the reviewer. We have now removed the correlation between the two indices from results and moved it to methods. The sentence in methods now reads:** **“NDWI min (the minimum NDWI min recorded over three years) and the range of NDWI (max NDWI − min NDWI) were both correlated with NDWI mean (r = 0.96 and r = -0.57 respectively, S1 appendix). We decided to use NDWI mean as a more reliable index of the long term moisture dynamics of peatlands” (lines 217-221).**

**For the reasons mentioned in the last part of the previous sentence we kept NDWI mean for our models but we consider relevant that the absolute driest peatlands (lowest NDWI values) are those more likely to be occupied.**

Discussion

Line 315: Snowy Plovers

**R: Corrected**

Line 320: Oystercatcher

**R: Corrected**

Line 325: Plovers

Line 330: similarly-sized

**R: We re-wrote the sentence and it now reads: “Other plovers of similar size” (line 333)**

Line 340: Dunlins and Wilson’s Snipes

**R: Corrected.**

Line 341: “…shorebirds that show no defense against cattle like the Diademed Plovers” – wording is somewhat unclear. I suggest either omitting, or rewording as “shorebirds that, like the Diademed Plovers, show do defense…”

**R: We followed the reviewer’s suggestion. The sentence now reads: “…****shorebirds that, like Diademed Plovers, show no defense against cattle but at sites where livestock graze year-round.” (lines 344-345)**


Lines 369-371: Which could lead to a positive association with NDWI in drier years – perhaps important to note.

R: **We rewrote the sentence and it now reads: “In drier years, small peatlands may become unsuitable (no running water) and peatlands that were too humid for Diademed Plovers in 2021–2022 may become suitable, reversing the relation with NDWI” (lines 373-375)**
